# Explainable AI-based clinical decision support system for flatfoot classification using deep learning

**DOI:** 10.3389/fdgth.2026.1852739

**Published:** 2026-07-29

**Authors:** V. Harsha Vardhan, S. G. Rahul, T. M. Amirthalakshmi, K. Venkatasubramanian

**Affiliations:** 1 Department of Electronics and Communication Engineering, Amrita School of Engineering, Amrita Vishwa Vidyapeetham, Chennai, India; 2 École Centrale School of Engineering, Department of Electrical and Computer Engineering, Mahindra University, Hyderabad, India

**Keywords:** explainable AI, interpretable machine learning, local binary pattern, monte carlo cross validation, pes planus, ResNet101V2, RMSprop

## Abstract

**Introduction:**

Flatfoot, also known as pes planus, is a deformity of the foot characterized by a decrease or absence of the medial longitudinal arch, which may lead to postural and locomotion abnormalities.

**Methods:**

This study presents a comparative analysis of handcrafted feature-based machine learning (LBP with Random Forest, Decision Tree, Logistic Regression) and deep learning models (InceptionResNetV2, ResNet101V2, DenseNet201, DenseNet169, InceptionV3, Xception) for flatfoot classification. Monte Carlo cross-validation was employed with subject-wise splitting. SHAP and Grad-CAM were used for explainability.

**Results:**

Random Forest achieved 70% accuracy with LBP features. ResNet101V2-RMSprop achieved 92.86% ± 2.82% mean accuracy and 98.21% best single-run accuracy. Deep features with Decision Tree achieved 97% accuracy, 95% recall, and 100% specificity.

**Discussion:**

SHAP and Grad-CAM confirmed that the model focuses on clinically relevant regions: the medial longitudinal arch and calcaneus for pes planus, and the talus-navicular region for normal feet. The hybrid approach is suitable for clinical screening.

## 1 Introduction

Pes planus, commonly known as flatfoot, is a chronic foot deformity characterized by partial or complete loss of the medial longitudinal arch, leading to altered biomechanics, gait abnormalities, and potential long-term musculoskeletal complications if left untreated (1). Clinical diagnosis traditionally relies on weight-bearing lateral foot x-rays, where angular measurements such as the calcaneal inclination angle (<18°) or Meary's angle serve as primary diagnostic criteria (2). However, manual measurement is subject to inter-observer variability and lacks standardization across clinical settings. These limitations motivate the need for an automated, interpretable deep learning system that can provide consistent classification, highlight decision-relevant image regions, and serve as a second opinion tool for clinicians.

### 1.1 Deep learning for pes planus using x-Ray images

Recent studies by Dogan et al. have explored both computational and clinical methods for analyzing and detecting pes planus. A hybrid CNN and Vision Transformer model, along with ensemble learning, was suggested for automatically detecting pes planus from weight-bearing x-ray images. This model achieved 97.4% sensitivity, 96.4% specificity, and 96.8% F1-score, demonstrating high diagnostic reliability (3). Hongwei Li et al. presented the mirroring weights-and-structure-determination neural network (MWASDNN) for diagnosing juvenile flatfoot using video content (4). Gul et al. identified how feature selection improves x-ray flatfoot image classification performance while reducing computational load, with SVM (cubic kernel) effectively classifying between normal and flatfoot conditions (5). Danaci et al. worked on pes planus diagnosis through x-ray images using machine learning features and deep learning methods, achieving 97% accuracy with XGBoost after feature selection (6). Kokulu et al. proposed a transfer learning framework using dilation filtering and DenseNet-201, achieving 98.02% classification accuracy (7). Wu et al. incorporated attention mechanisms and Grad-CAM for interpretability in foot type diagnosis (8). Koo et al. developed a deep learning tool to improve reproducibility of radiographic angle measurement (9). A modified version of the MWASDNN was later proposed for improved pattern classification of flatfoot (10). Kimyeonho and Kim achieved test accuracies around 84% using transfer learning on augmented foot images (11). Alsaidi and Moria developed a foot image arch angle measurement system for flatfoot severity-level detection based on alignment measuring (12).

### 1.2 Alternative sensing modalities for foot type classification

Beyond x-rays, researchers have explored alternative sensing modalities. Sankaran et al. developed a sensor-based system using piezoelectric sensors in footwear to measure plantar pressure and support personalized corrective footwear (13). Ardhianto et al. developed a YOLOv4-based object detection model to automatically identify left and right footprints from plantar pressure images, achieving over 99% detection accuracy (14). Zhao et al. used deep learning for plantar pressure images, with a ResNet-50 model achieving 82.6% accuracy for foot type classification (15). Haripriya et al. constructed a foot scanner with a CNN model trained on foot images for automated arch classification (16). Ivanov et al. developed a smart insole with a 1D-CNN model achieving 99.26% accuracy in distinguishing normal, cavus, and planus feet, though on a limited demographic of 80 subjects (17). Pelok et al. introduced a deep learning framework for semantic segmentation using RGB video data to assess physiotherapy exercises for flatfoot rehabilitation (18). Sawangphol et al. explored foot arch classification using machine learning-based image classification approaches (19). Sen and Das developed an in-house early detection system for flatfoot and high ankle foot using image processing techniques (20). Garcia-Estrada et al. concluded that foot plantar deformations are categorized by their effect on the footprint, with flatfoot and supinated foot being the most common (21).

### 1.3 Clinical and biomechanical context

Research by Smyth et al. explains how Adult-Acquired Flatfoot Deformity (AAFD) progresses through four recognizable stages, from posterior tibial tendon tenosynovitis to fixed deformity with ankle involvement (22). Pita-Fernandez et al. reported pes planus prevalence rates ranging between 20% and 37% in random populations (23). Bernasconi et al. highlighted subtalar arthroereisis as a minimally invasive surgical option for correcting symptomatic flatfoot deformities (24). The static footprint evaluation method by Uluoz et al. for detecting pes planus used the Harris mat with Staheli index, Chippaux-Smirak index, and Grivas Classification System (25). Buldt et al. discovered substantial variations between pressure and force parameters between planus and cavus feet (26). Carr et al. evaluated pediatric pes planus, discussing common misconceptions and the absence of a standardized classification process (27). Gutierrez-Vilahú et al. analyzed podometric tools for footprint classification in Down syndrome patients, showing excellent reliability for identifying flat feet (28). Ueki et al. examined pathology and orthotic management of flexible flatfoot in children, stressing radiographic and ultrasonographic evaluation for treatment planning (29). Caravaggi et al. analyzed skin-marker-based models for measuring medial longitudinal arch movement, identifying significant error differences across models (30). Uden et al. contributed to understanding foot development in children, which is crucial for clinical assessments and interventions (31). Vijayakumar et al. analyzed foot arches using a newly designed podoscope, grading the severity of pes planus and pes cavus (32). Jung et al. proposed decision tree-based foot orthosis prescription for patients with pes planus (33). A comparative analysis of different optimizers for deep learning-based image recognition was presented by Postalcioglu (34).

### 1.4 Research gaps identified

- Existing studies do not provide a unified comparison of handcrafted feature-based ML models, standalone deep learning models, and hybrid deep feature and ML approaches for pes planus classification.
- Machine learning classifiers have rarely been tested on deep features taken from CNN models. This leaves the effectiveness of hybrid pipelines largely unstudied.
- Explainable AI techniques like SHAP-based feature attribution are not widely used for interpreting pes planus classification. This limits clinical transparency and trust.
- The effect of different optimizers on deep learning performance and their impact on downstream hybrid ML classification has not been systematically studied in the existing literature.

### 1.5 Major contributions of this study

The current study conducted a novel comparative analysis of deep learning models, handcrafted feature extraction, and deep feature extraction followed by machine learning classifier training for pes planus foot classification. To further improve diagnostic accuracy, feature concatenation from several deep learning models was investigated.

(a) Handcrafted Feature-Based Machine Learning: To capture important structural details of the foot, texture features were extracted using the Local Binary Pattern (LBP) method. Machine Learning (ML) algorithms, including Random Forest (RF), Decision Tree (15), and Logistic Regression (LR), were then used to classify these features.
(b) Deep Learning-Based Classification: Deep learning models, namely InceptionResNetV2, ResNet101V2, DenseNet201, DenseNet169, InceptionV3, and Xception, were trained on an augmented dataset. The models directly classified images into normal or pes planus categories.
(c) Deep Feature Extraction & ML Classification: To further improve accuracy, deep features were extracted from each trained deep learning model. These extracted features were then trained with ML classifiers.
(d) Incorporation of Explainable AI: Explainable AI techniques, including SHAP decision plots, heatmaps, and feature importance bar plots, are incorporated to provide interpretability by visualizing the regions or features most influential in classification decisions (Normal vs. Pes Planus foot). This enhances transparency, increases clinician trust in AI-based diagnosis, and validates computational findings against established clinical indicators of flatfoot.

## 2 Materials and methods

### 2.1 Dataset description

**Figure 1:**
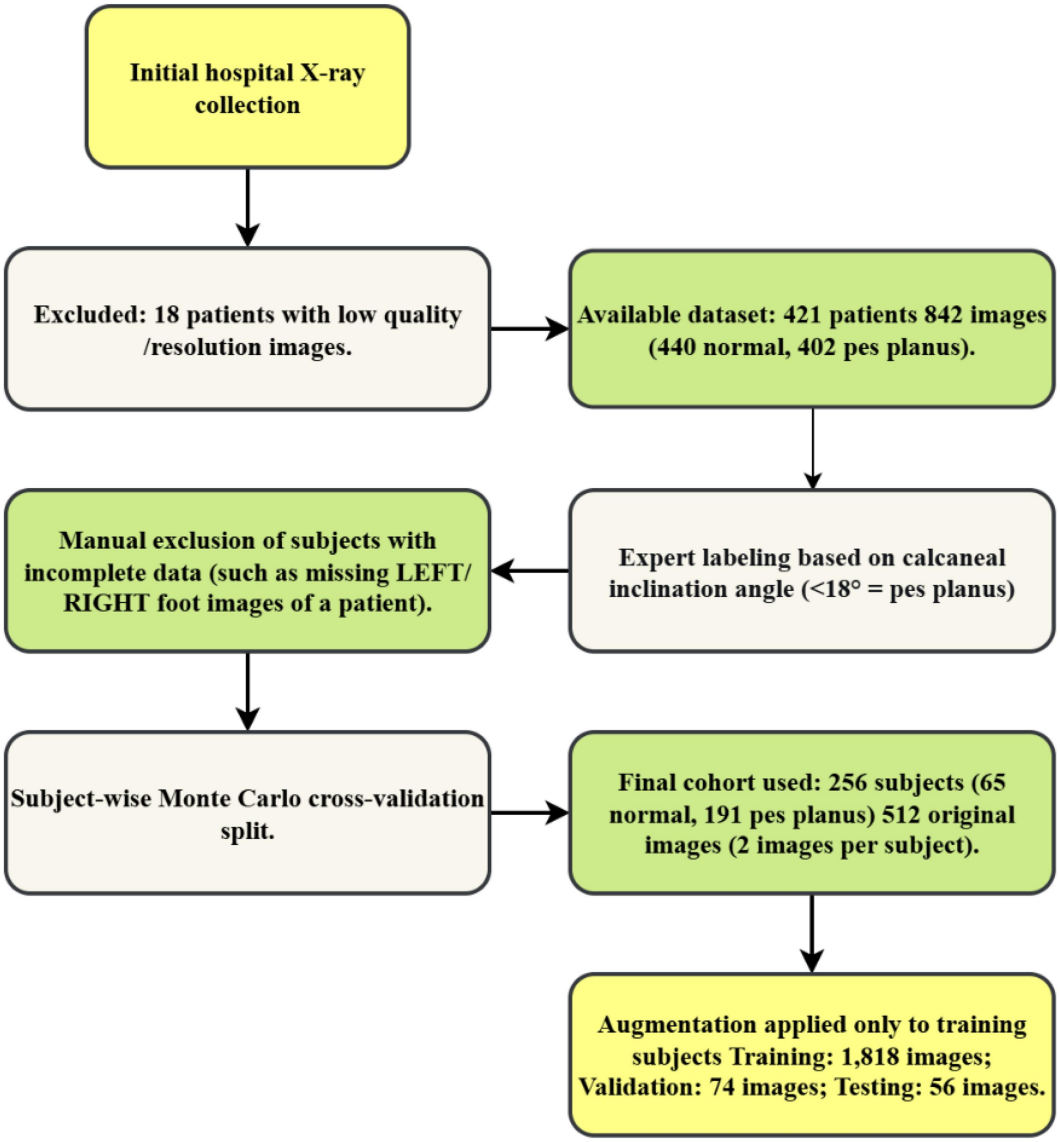
Flowchart of subject and image selection.

In this study, a specialized pes planus x-ray dataset was developed using weight-bearing lateral foot radiographs collected from the Radiology Department of Elazığ Fethi Sekin City Hospital in Turkey. The images were taken from patients aged 14–47 who either had routine pre-military health screenings or were suspected of having flatfoot. The Ethics Committee of Fırat University in Turkey approved the image collection. All radiographs were taken using a Philips dual-detector digital x-ray system at 65 kV and 6.3 mAs, and the images were saved in .JPG format. Patients with known neurological disorders, recent orthopedic injuries, or a history of lower limb orthopedic surgery were excluded for this study. In the collected pes planus dataset, x-ray images of 18 patients with low quality and resolution were discarded, resulting in 842 high-quality x-ray images from 421 patients. The dataset was labeled by two experienced radiologists based on the calcaneal inclination angle. A third specialist radiologist performed the post-labeling by re-examining the conflicting examples. In the final classification, x-ray images with a calcaneal angle below 18° were classified as pes planus, while those with angles of 18° and above were labeled as normal. After applying subject-wise Monte Carlo cross-validation splits and excluding subjects with incomplete data, the final cohort comprised 256 subjects (65 normal, 191 pes planus) with 512 original images (2 images per subject). Figure 1 presents a flowchart summarizing the subject and image selection process.

**Figure 2:**
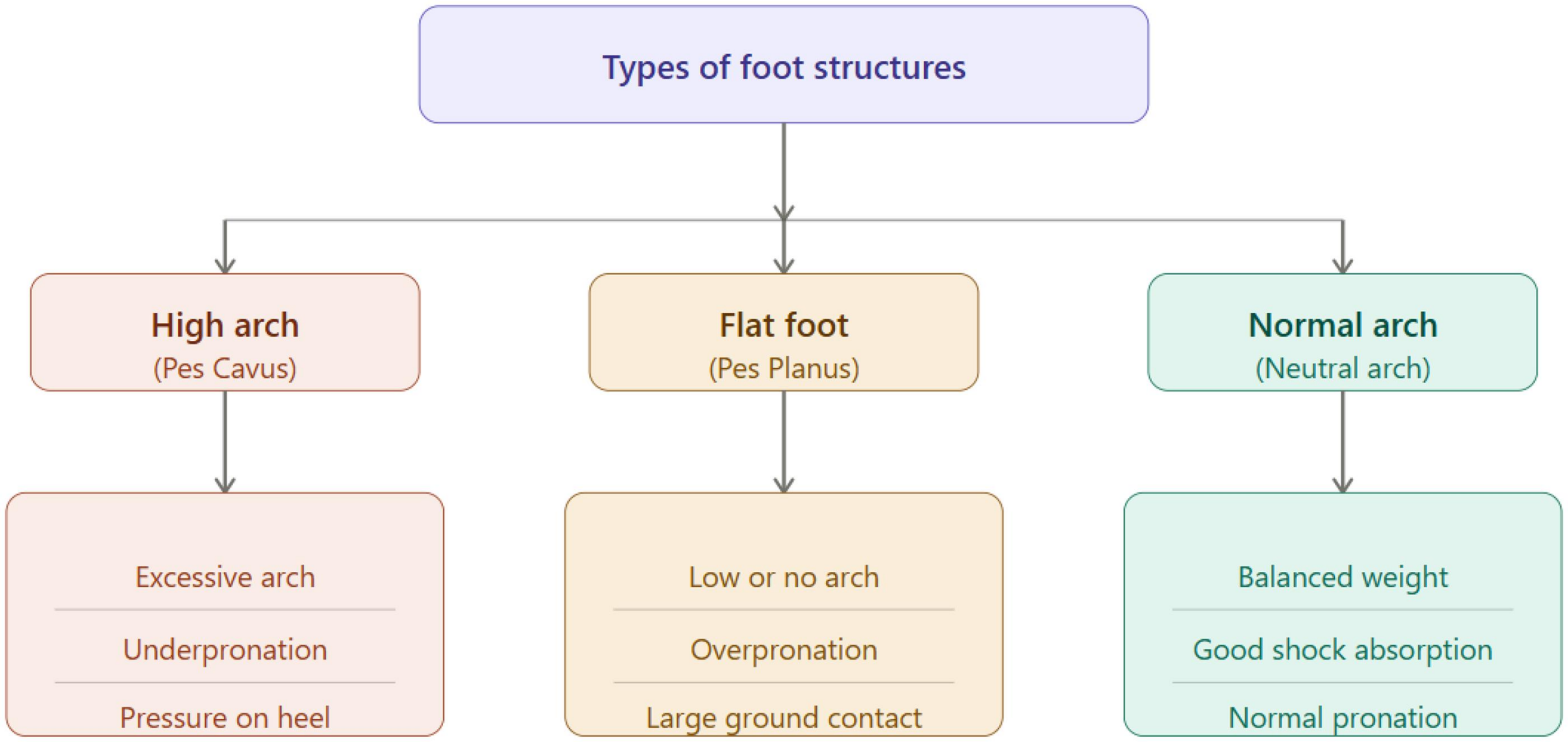
Types of foot structures.

Figure 2 shows three main types of foot arches are classified based on anatomy and biomechanics of foot structure: high arch, normal arch, and flat foot (19).

**Figure 3:**
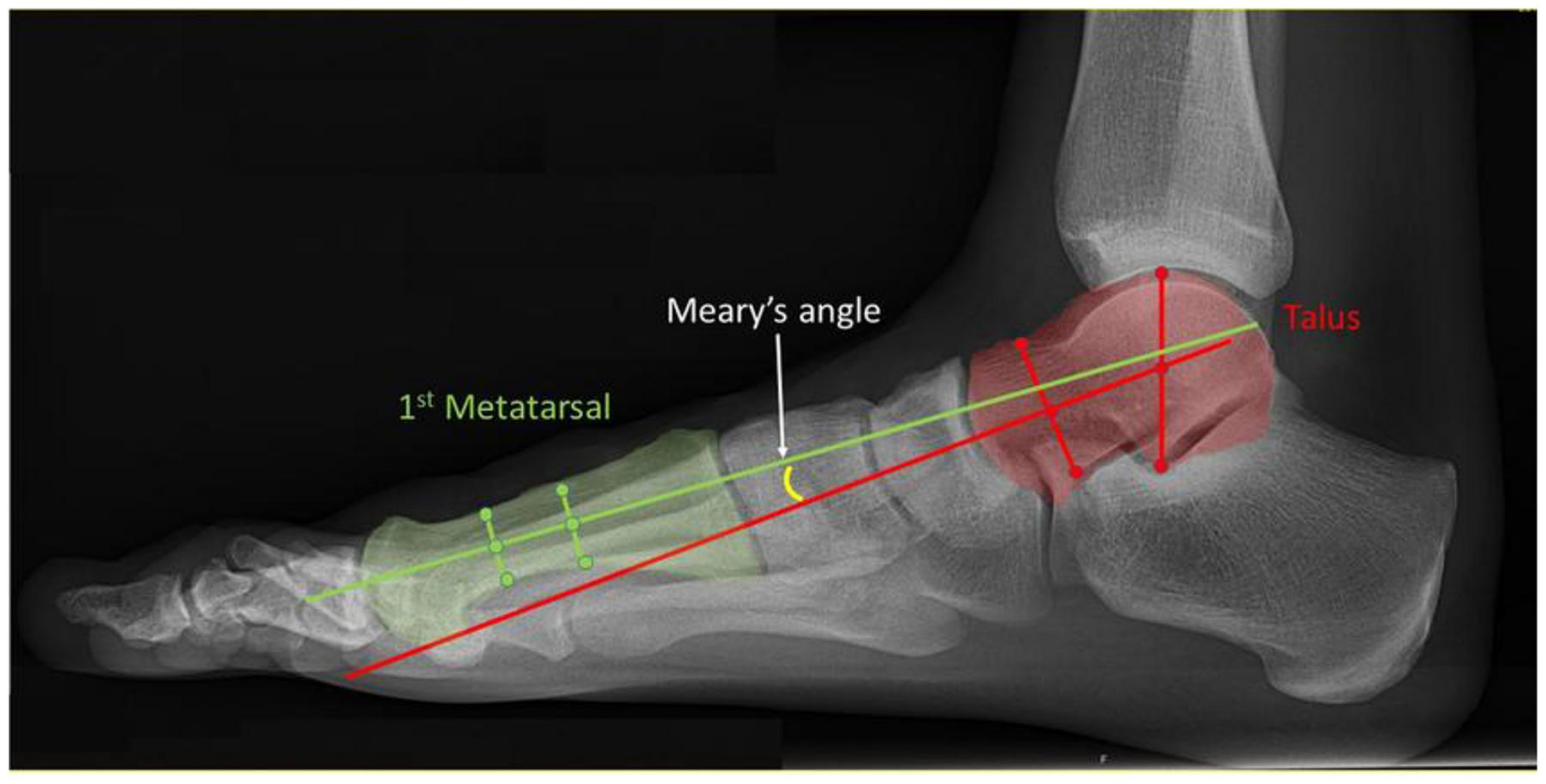
Meary's angle: the angle between the longitudinal mid-talar axis and the longitudinal first metatarsal axis.

Meary's angle is shown here for anatomical reference only; it was not used as the labeling criterion. In a normal weight-bearing foot, the axes are aligned (angle = 0°). Calcaneal inclination angle <18° is considered pes planus (2) (Figure 3). Table 1 shows how the dataset was split based on the subjects' samples.

**Table 1:** Dataset subject-wise split following Monte-Carlo CV (MC-CV).

**Split**	**Subjects**	**Images**	**Augmentation**
Training	191	1,818	Yes (training subjects only)
Validation	37	74	No
Testing	28	56	No
Total	**256**	**1,948**	–

Bold values indicate the highest values in each column.

Table 2 shows class-wise distribution of images at each Monte Carlo CV split.

**Table 2:** Class-wise subject distribution per run.

Class	Training	Validation	Testing	Total Subjects
Normal foot	49	10	6	65
Pes planus foot	142	27	22	191
Total	191	37	28	256

### 2.2 Monte carlo cross-validation

**Figure 4:**
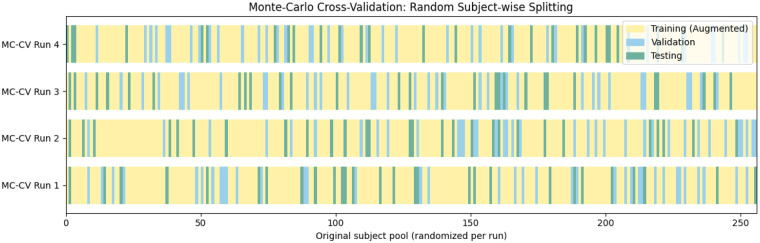
Monte-Carlo cross-validation: random subject-wise splitting.

The dataset comprised 256 subjects (65 normal-foot and 191 pes-planus), with two original images per subject. A stratified subject-wise Monte Carlo cross-validation strategy was employed, as in Figure 4. In each run, 49 normal-foot and 142 pes-planus subjects were allocated to training, while the remaining subjects were used for validation and testing. Data augmentation was applied offline and exclusively to training subjects, generating ten augmented samples per original normal-foot image and three per original pes-planus image. Consequently, the training set size was fixed at 1,818 images across all runs, while validation and test sets contained only original images (74 and 56 images, respectively). In each Monte Carlo run, preprocessing {resizing to 500 × 500 pixels and pixel intensity normalization to the [(0,1)] range} and feature scaling (for LBP features and deep features) were performed independently using only the training subjects to prevent any data leakage from validation or test sets. Data augmentation was applied offline to the training subjects prior to the experiment, with augmented images stored in a separate directory (AUGMENTED_DIR). For each original normal-foot image, ten augmented samples were generated; for each original pes planus image, three augmented samples were generated. A fixed random seed (42) was used for all randomization processes, including subject split selection and model weight initialization. For each Monte Carlo run, a unique seed (SEED + run + 151) was used to ensure independent splits while maintaining reproducibility. The same preprocessing and scaling parameters computed from the training set were then applied to the validation and test sets without refitting.

### 2.3 Methods

**Figure 5:**
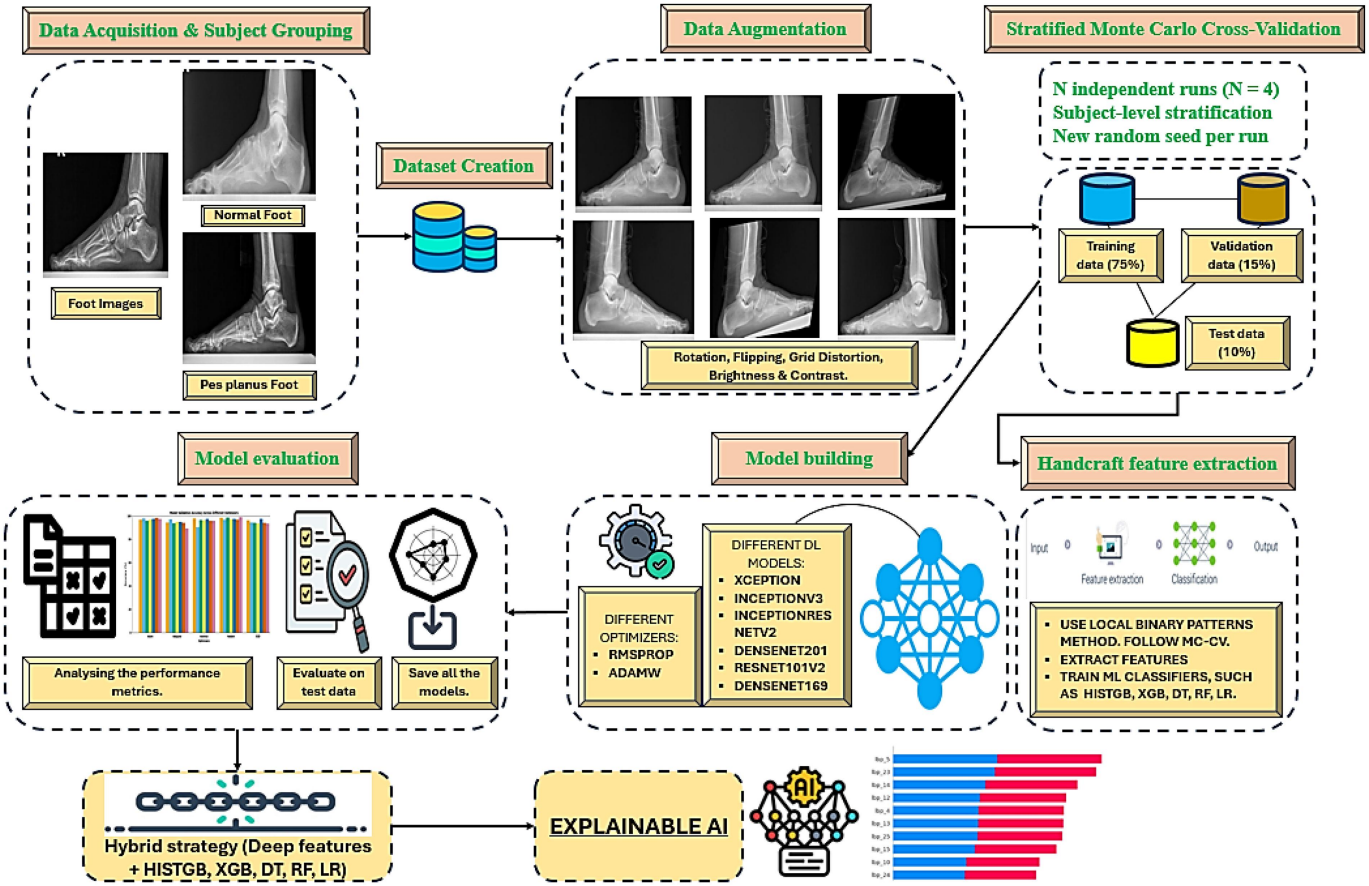
Research workflow and methodology.

The current research study's proposed workflow is depicted in Figure 5, including dataset preparation, data augmentation, cross-validation, deep learning model development, handcrafted feature extraction, hybrid classification, model evaluation, and explainable AI analysis.

#### 2.3.1 Handcrafted feature extraction

Local Binary Pattern (LBP) captures local details from images. The method compares the intensity of each pixel to its neighboring pixels within a set radius. If a neighbor's intensity is equal to or greater than the center pixel, it receives a value of one; otherwise, it gets a zero. This is mathematically represented as (Equation 1).

$$v(q)=\{\begin{array}{c} \;1,\;\;\mathrm{if}\;q\geq 0\\ 0,\;\;\mathrm{if}\;\;q\;\;<\;0 \end{array}$$ (1)

These binary values are arranged in a sequence and converted into a decimal number. This number represents the texture pattern at that specific location. In detecting pes planus, LBP play a crucial role. This condition alters the structure of the medial longitudinal arch of the foot. These changes show up as differences in bone alignment, cortical thickness, and trabecular texture in x-ray images. While raw grayscale values might overlook these small variations, LBP captures them well by encoding the texture of edges, ridges, and smooth areas. This improves the visibility of clinically important features, allowing computational models to differentiate more reliably between normal and flatfoot conditions. The LBP operator is mathematically given as (Equation 2):

$$LBP_{\left\{P,R\right\}}=\;\overset{\left\{P-1\right\}}{\underset{\left\{p=0\right\}}{\sum }}\;v(N_{i}-\;C_{i})\cdot 2^{p}$$ (2)

where P is the number of neighbors, R is the radius, Ci is the center pixel intensity, and Ni are the neighboring pixel intensities. This operation results in a transformed image where each pixel encodes meaningful local textural patterns.

**Figure 6:**
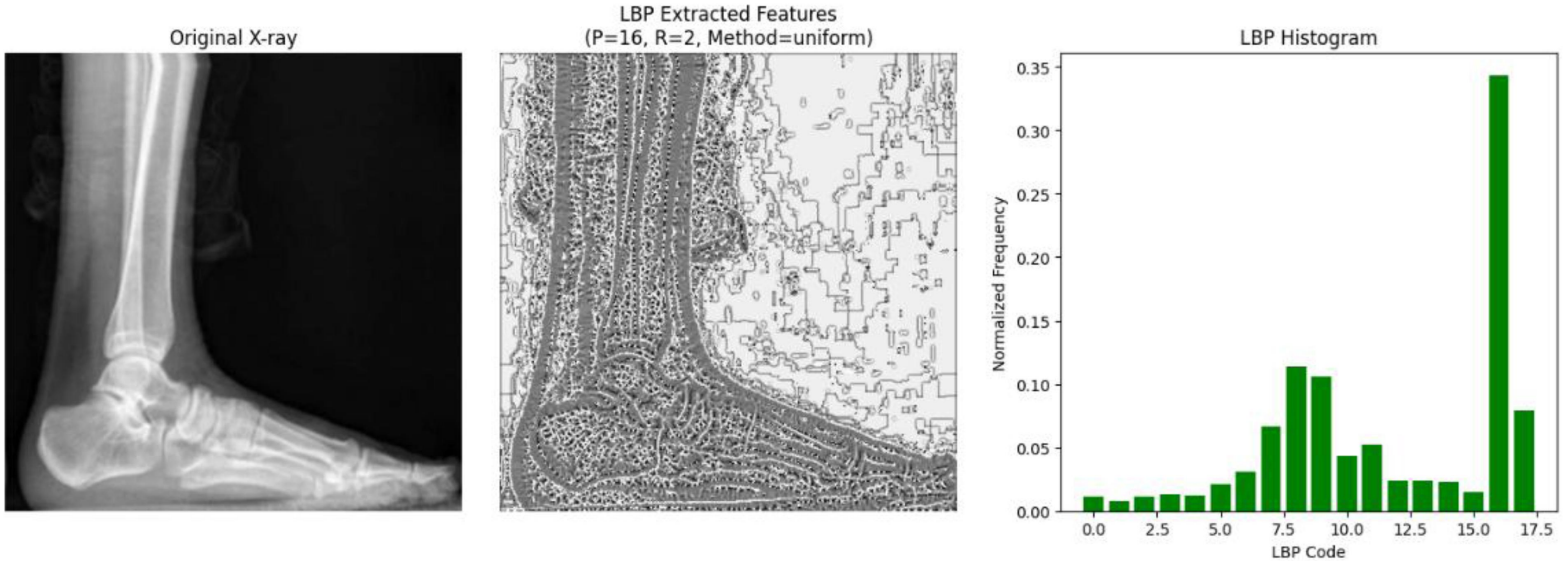
Feature extraction through local binary pattern method.

The chosen parameters of *P* = 16 neighbors and R = 2 radius with “uniform” method works well for medical imaging. A larger neighborhood size gives more texture information without being overly sensitive to noise. The uniform pattern cuts down on the number of possible LBP codes. This leads to more compact and rotation-invariant feature representations. This balance makes sure that fine structural variations in x-rays stand out while avoiding unnecessary complexity. Once the LBP features are extracted, they are summarized using a histogram that represents the frequency distribution of the encoded texture patterns across the image. As depicted in Figure 6, each bin in the histogram represents a specific LBP code, and the height of the bin indicates how often that texture pattern appears in the x-ray.

After extracting Local Binary Pattern (LBP) features from pes planus and normal foot x-ray images, these features are standardized to have a mean of zero and a variance of one. This procedure prevents bias toward higher values and ensures that each feature contributes equally. The standardization formula is shown in Equation 3:

$$F^{\prime }=\frac{F\;-\;\mu }{\sigma }$$ (3)

The original feature is denoted by F, the mean by µ, and the standard deviation by *σ*. Standardization is important for algorithms like Logistic Regression since they can be sensitive to scale. It also improves convergence and stability during model training. The Random Forest classifier is an ensemble of decision trees. It uses n_estimators = 100 in this study. Its strength comes from combining several weak learners to create a strong, robust model that can capture complex non-linear relationships within the LBP feature space. Random Forest reduces overfitting and provides implicit measures of feature importance. The Decision Tree classifier is simpler yet interpretable. It divides features based on criteria like Gini impurity or information gain. Each node represents a decision based on LBP histogram values. Logistic Regression uses a probabilistic framework to estimate the likelihood of a patient having pes planus. With proper regularization, it avoids overfitting while staying computationally efficient.

#### 2.3.2 Performance assessment of multiple deep learning models on the dataset

To keep training consistent across all chosen architectures, we used a uniform set of hyper parameters. All models were trained with an input image size of 500 × 500 pixels and a batch size of 32. This setup was able to balance computational efficiency and stable gradient updates. We applied dropout regularization with probabilities of 0.5 and 0.3 at different layers to reduce overfitting by preventing neurons from relying too much on one another. This change improved the models' ability to generalize. Additionally, early stopping was used with a patience threshold of 10 epochs to track validation performance and stop training when further improvement seemed unlikely. This approach avoided unnecessary computation and lowered the risk of over fitting.

##### 2.3.2.1 InceptionV3

**Figure 7:**
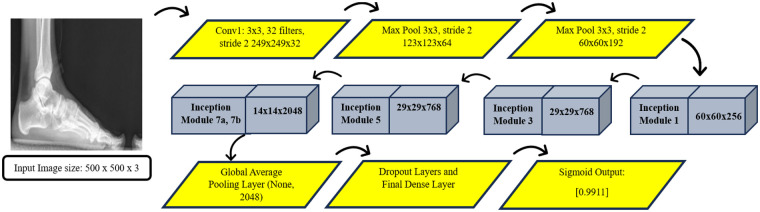
Inceptionv3 architecture.

Inception V3 model (Figure 7) takes an input image with a size of 500 × 500 × 3 and applies convolution (output: 250 × 250 × 32) and max pooling (output: 124 × 124 × 64), then another layer of max pooling (output: 61 × 61 × 192). The features are then processed through multiple Inception modules which narrow down the spatial dimensions, while increasing depth, resulting in a feature map of size 61 × 61 ×  2,048. This is then followed by global average pooling to obtain a 2,048 dimension vector, dropout layers, and a dense layer, with a final sigmoid activation that outputs the probability for normal vs. pes planus foot classification. (Table 3).

**Table 3:** Training configurations of deep learning models.

Model	Image size	Dropout values	Learning rate	Batch size	Epochs (per run)	Early stopping (patience)
InceptionResNetV2	500 × 500	0.5, 0.3	1 × 10^−4^	8	50 (max)	10
ResNet101V2	500 × 500	0.5, 0.3	1 × 10^−4^	8	50 (max)	10
InceptionV3	500 × 500	0.5, 0.3	1 × 10^−4^	8	50 (max)	10
Xception	500 × 500	0.5, 0.3	1 × 10^−4^	8	50 (max)	10
DenseNet201	500 × 500	0.5, 0.3	1 × 10^−4^	8	50 (max)	10
DenseNet169	500 × 500	0.5, 0.3	1 × 10^−4^	8	50 (max)	10

##### 2.3.2.2 ResNet101V2

**Figure 8:**
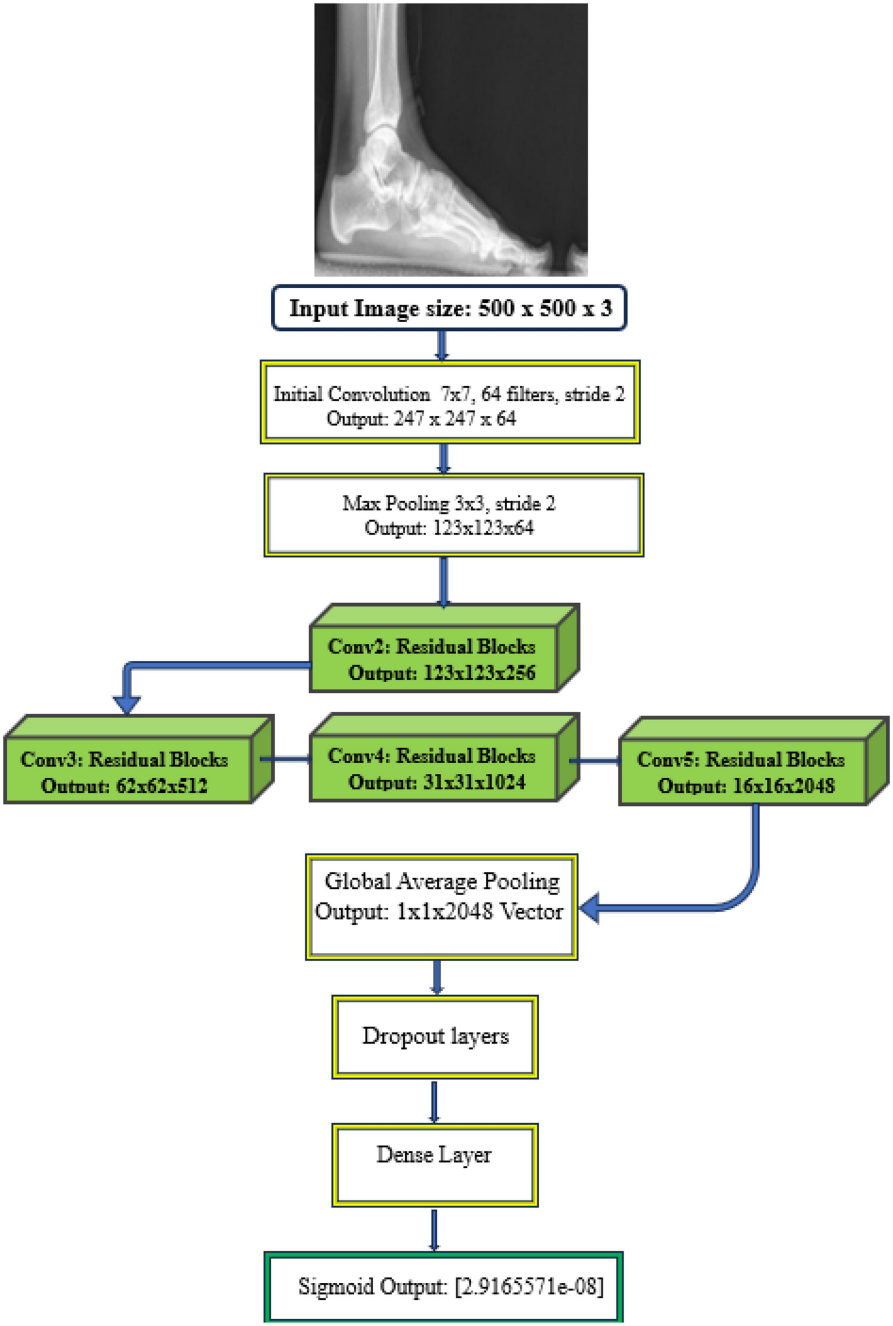
ResNet101V2 architecture.

The ResNet101V2 architecture as depicted in Figure 8 begins with convolution and max pooling. Residual blocks in Conv2 to Conv5 stages capture low-level to high-level features. Global average pooling converts the feature map to a 2,048-dimensional vector. This vector is processed by dropout and fully connected dense layers. A sigmoid function in the output layer produces the final classification.

##### 2.3.2.3 DenseNet201

**Figure 9:**
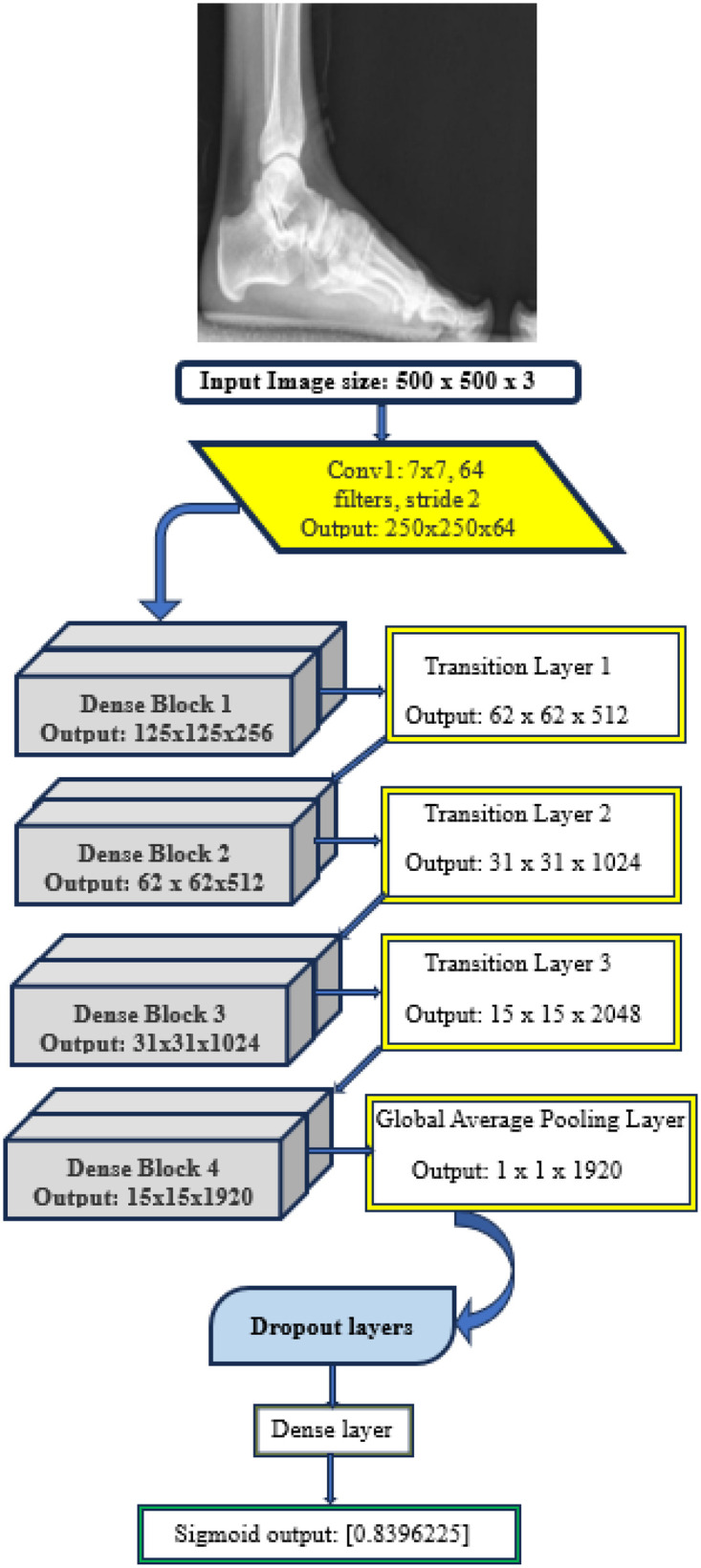
Densenet201 architecture.

The DenseNet201 architecture (Figure 9) begins with convolution and max pooling. Features are propagated through dense blocks with 6, 12, 32, and 48 layers. Transition layers compress and downsample the feature map size. Global average pooling produces a sparse vector from the final feature map. Dropout layers and a sigmoid activation prevent overfitting and enable classification.

##### 2.3.2.4 Focal loss

Focal loss was chosen over standard binary cross-entropy to address the inherent class imbalance commonly present in medical imaging datasets like Pes Planus. By introducing a modulating factor (1−pt)*^γ^* with *γ* = 2.0, it down-weights the loss contribution from easy, well-classified samples and forces the model to focus learning on hard, misclassified examples. The alpha parameter of 0.75 further penalizes false negatives more heavily, which is clinically critical in a diagnostic setting where missing a Pes Planus case carries greater consequence than a false alarm.

##### 2.3.2.5 Channel attention mechanism

Channel attention was incorporated into the architecture to enable the model to selectively emphasize the most diagnostically informative feature maps generated by the backbone. The squeeze-and-excitation-style mechanism applied here compresses spatial information via GlobalAveragePooling, then uses a two-layer dense bottleneck with ReLU and sigmoid activations to learn inter-channel dependencies and produce a recalibration vector. This recalibration multiplicatively rescales the feature maps before the classification head, effectively acting as a learned feature selector. By allowing the model to adaptively weight channels based on their relevance to the target condition, channel attention improves both convergence stability and generalization, especially on a relatively small medical dataset where overfitting to irrelevant features is a genuine concern.

#### 2.3.3 Optimizers

Two optimizers were compared in this study. AdamW extends Adam by decoupling weight decay from gradient updates, improving regularization and generalization. RMSprop modifies learning rates by dividing the gradient by a decaying average of its squared values, making it suitable for non-stationary objectives. Both optimizers were used with a learning rate of 1 × 10⁻^4^ (34).

#### 2.3.4 Deep feature extraction from DL models and classifiers

After training the CNN model, we extract deep features from the last fully connected layer. This layer captures important information for classifying pes planus. We extract these features for both the training datasets, which include the original and augmented balanced data, and for the independent test dataset using the same preprocessing steps. These extracted features are standardized and then used to train several machine learning classifiers, such as Random Forest, Decision Tree, Logistic Regression, Histogram-based Gradient Boosting, and XGBoost. We use GridSearchCV with 5-fold cross-validation to optimize hyperparameters (Table 4). We evaluate the best models on the test set using classification metrics and confusion matrices. The pseudocode of the deep feature-based ML classification process is presented in Algorithm 1.

**Table 4:** Best parameters searched using GridsearchCV library, with cross validation=5.

Classifier	Hyperparameters searched
Random Forest	n_estimators: {200, 400}
	max_depth: {None, 20}
	min_samples_leaf: {1, 2}
Decision Tree	max_depth: {None, 10, 20}
	min_samples_leaf: {1, 2}
Logistic Regression	C: {0.1, 1, 10}
	max_iter: 3,000
Histogram-Based Gradient Boosting	max_depth: {None, 10}
	learning_rate: {0.05, 0.1}
	max_iter: {200, 400}
XGBoost	n_estimators: {200, 400}
	max_depth: {3, 5}
	learning_rate: {0.05, 0.1}
	subsample: {0.8, 1.0}
	colsample_bytree: {0.8, 1.0}

Algorithm 1 **The pseudocode of the deep feature–based ML classification process.**

Input:

 Training image set I_train = I1, I2, … , IN}

 Test image set I_test = {J1, J2, … , JM}

 Trained CNN model M

Output:

 Classification performance metrics P

Load the trained CNN model M.

Construct the feature extractor F by removing the final classification layer from M.

For each image Ii ∈ I_train, preprocess the image and extract the deep feature vector

fi = F(Ii).

Form the training feature matrix X_train and label vector y_train.

For each image Jj ∈ I_test, preprocess the image and extract the deep feature vector

fj = F(Jj).

Form the test feature matrix X_test and label vector y_test.

Standardize X_train using a StandardScaler and apply the same scaling parameters to

X_test.

Define the classifier set

C = {Random Forest, Decision Tree, Logistic Regression, HistGB, XGBoost}.

For each classifier c ∈ C, perform hyperparameter optimization using GridSearchCV with

5-fold cross-validation, train the optimized classifier on X_train, predict labels on

X_test, evaluate the classification performance, and save the trained model.

#### 2.3.5 Explainable AI (XAI) methodology with SHAP

To interpret model predictions, SHAP (SHapley Additive exPlanations) was applied to the best-performing deep learning models. The KernelExplainer was used with a background sample of 50 randomly selected training images to approximate feature importance. For each test image, SHAP values were computed for all deep features extracted from the penultimate layer. Decision plots visualize how individual features push the prediction toward normal or pes planus classes. Heatmaps aggregate SHAP values across multiple test samples to reveal class-consistent feature contributions. Feature importance bar plots show mean absolute SHAP values per feature across each class. All SHAP visualizations were generated using the SHAP Python library (version 0.41.0).

SHAP was chosen over spatial attention methods like Grad-CAM because it provides global feature attribution independent of model architecture and allows comparison across different deep learning backbones. However, we acknowledge that Grad-CAM could offer more intuitive spatial heatmaps; this is a planned extension.

### 2.4 Evaluation metrics

Model performance was evaluated using standard classification metrics derived from the confusion matrix (true positives TP, true negatives TN, false positives FP, false negatives FN): accuracy, sensitivity (recall), specificity, precision, F1-score, and area under the ROC curve (AUC).

## 3 Results

### 3.1 LBP

The performance of machine learning classifiers using LBP features is shown in Table 5. Among the models evaluated, Random Forest achieved the highest test accuracy at 72%, showing its strong ability to capture texture patterns. XGBoost came next with 70% accuracy, while Decision Tree and HistGradient Boosting had similar performance at 69%. Logistic Regression had a lower accuracy of 66%, which suggests that LBP features have limited linear separability. To sum up, ensemble methods performed better than single and linear classifiers.

**Table 5:** Test accuracy of Various machine learning classifiers on the dataset (values in %).

Model	Test Accuracy (%)
Random Forest	72%
Decision Tree	69%
Logistic Regression	66%
HistGB	69%
XGBoost	70%

**Figure 10:**
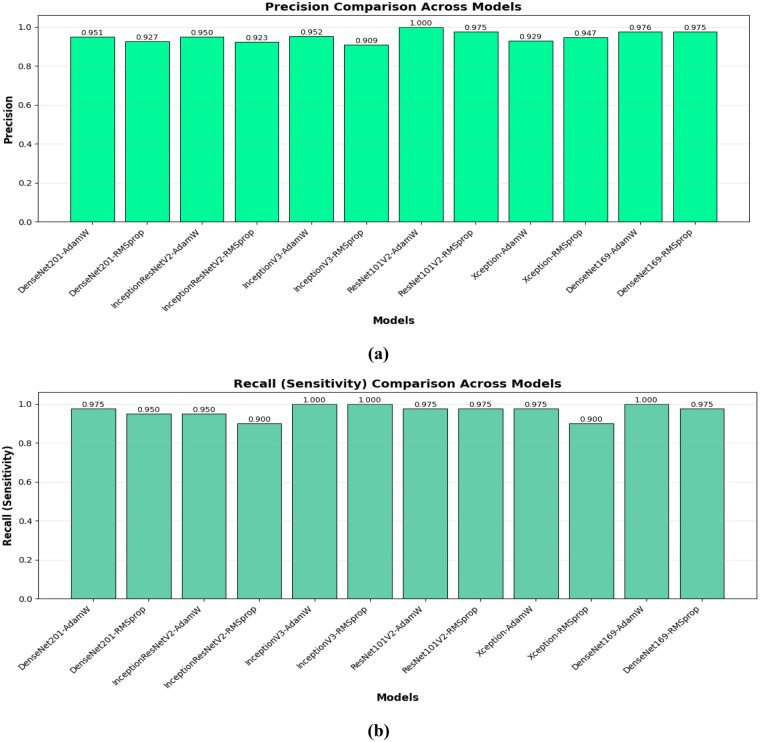

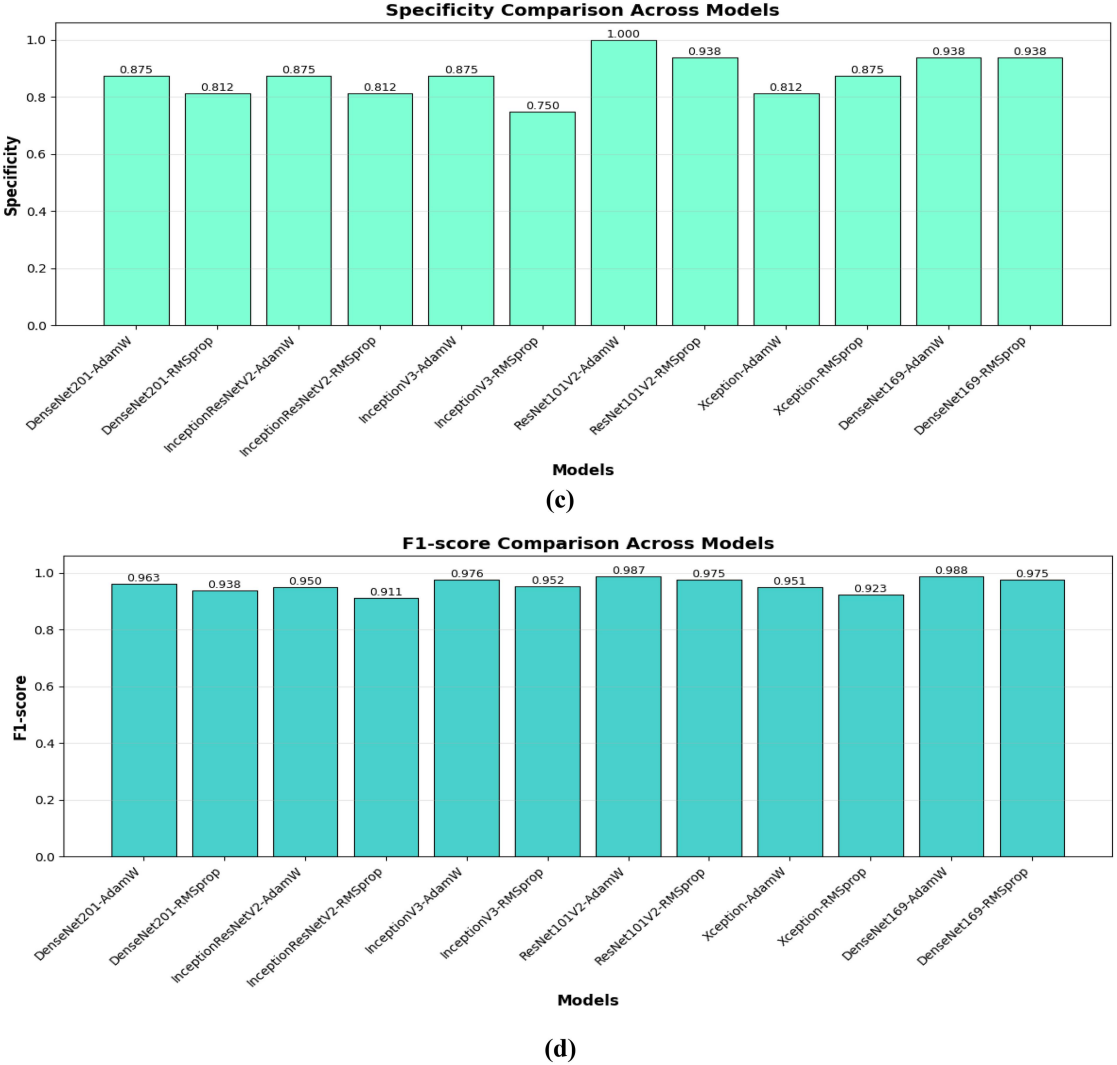
Performance metrics of classifiers trained with deep features. **(a)** Precision; **(b)** Recall; **(c)** Specificity; **(d)** F1-Score.

The evaluated model and optimizer combinations performed well in classifying Pes Planus and Normal foot types. There were noticeable differences in training convergence, validation stability, and feature representation. The performance of the deep learning models was evaluated using accuracy, AUC, precision, recall (sensitivity), specificity, and F1-score, as summarized in Table 6 and Figures 10a–d. Overall, models optimized with AdamW consistently showed better performance than those using RMSprop, indicating more stable convergence and improved generalization. Among all the architectures tested, DenseNet169-AdamW and ResNet101V2-RMSprop achieved the highest mean classification accuracy, exceeding 92%, along with strong AUC values close to 0.98. These models also displayed balanced precision and recall, leading to high F1-scores. This reflects their strength in handling both positive and negative classes.

**Table 6:** Test accuracy of Various DL models on the dataset (values in %).

Model	Accuracy (%)	AUC	Best Accuracy (%)	Run number
DenseNet201-AdamW	**91.52** **± 3.43**	**0** **.** **942**	94.64%	**1**
DenseNet201-RMSprop	**89.73** **± 2.32**	**0** **.** **937**	91.07%	**2**
InceptionResNetV2-AdamW	**89.29** **± 2.82**	**0** **.** **914**	92.86%	**1**
InceptionResNetV2-RMSprop	**85.27** **± 1.95**	**0** **.** **879**	87.50%	**2**
InceptionV3-AdamW	**91.07** **± 3.99**	**0** **.** **970**	96.43%	**3**
InceptionV3-RMSprop	**90.62** **± 2.32**	**0** **.** **956**	87.50%	**1**
ResNet101V2-AdamW	**89.29** **± 7.36**	**0** **.** **962**	98.21%	**1**
ResNet101V2-RMSprop	**92.86** **± 2.82**	**0** **.** **979**	96.43%	**2**
Xception-AdamW	**87.50** **± 3.34**	**0** **.** **933**	92.86%	**1**
Xception-RMSprop	**86.16** **± 2.64**	**0** **.** **907**	89.29%	**1**
DenseNet169-AdamW	**92.41** **± 4.44**	**0** **.** **967**	98.21%	**1**
DenseNet169-RMSprop	**86.16** **± 8.22**	**0** **.** **950**	96.43%	**2**

Bold values indicate the highest values in each column.

### 3.2 Evaluation of deep learning models

To quantify the reliability of the top-performing models, 95% confidence intervals (CIs) were calculated. ResNet101V2-RMSprop achieved a mean accuracy of 92.86% ± 2.82% (95% CI: 87.3%–98.4%). DenseNet169-AdamW achieved 92.41% ± 4.44% (95% CI: 88.0%–96.8%). Balanced accuracy (the average of sensitivity and specificity) for ResNet101V2-RMSprop was approximately 97.5%, indicating robust performance across both classes despite the moderate class imbalance in the original dataset. The two top-performing models showed comparable performance with no statistically significant difference based on overlapping confidence intervals.

In terms of precision, ResNet101V2-AdamW achieved a perfect score, indicating zero false positive predictions in the evaluated runs. Similarly, several models, including InceptionV3-AdamW and DenseNet169-AdamW, achieved a recall of 1.000. This shows their effectiveness in correctly identifying all positive samples, which is crucial in medical diagnostic tasks.

The specificity analysis showed noticeable differences between models. While ResNet101V2-AdamW achieved perfect specificity, some RMSprop-based models displayed lower specificity, suggesting a higher chance of false positive classifications. This highlights the need to consider specificity alongside recall when assessing diagnostic reliability.

In contrast, InceptionResNetV2-RMSprop and Xception-RMSprop recorded lower accuracies and F1-scores. This indicates limited ability to distinguish between classes under the selected training setup. However, their moderate AUC values suggest acceptable ranking performance, even with reduced classification consistency.

**Figure 11:**
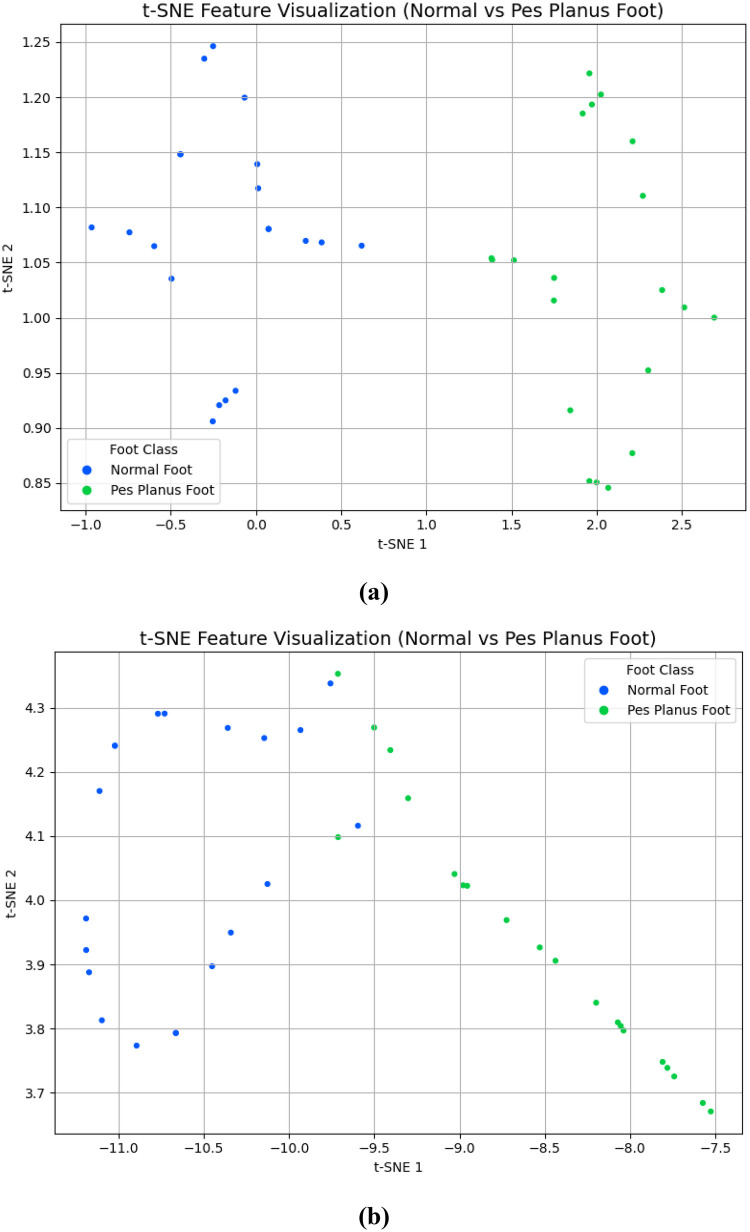
t-SNE plots of: **(a)** DenseNet169-RMSprop; **(b)** InceptionResNetV2-RMSprop.

Figure 11 shows the t-SNE visualization of deep features extracted using DenseNet169-RMSprop and InceptionResNetV2-RMSprop for normal and pes planus foot classes. The two-dimensional projections help us understand how well the specific features learned by each model separate the classes. Figure 11a shows that DenseNet169-RMSprop produces two well-separated clusters corresponding to normal and pes planus classes, with minimal overlap. In contrast, Figure 11b reveals that InceptionResNetV2-RMSprop exhibits significant class overlap and greater feature scattering, particularly for the pes planus class. These findings align with the performance metrics discussed earlier.

As evident from Table 7, DenseNet201-AdamW and ResNet101V2-RMSprop stand out as the strongest performers, consistently achieving 0.97 test accuracy, 0.95 precision and recall, and perfect 1.00 specificity across all five classifiers, suggesting these backbones extract highly discriminative features regardless of the downstream classifier used. DenseNet169-AdamW performs comparably well, with most classifiers hitting 0.97 accuracy, though DT drops slightly to 0.95. InceptionV3-AdamW shows more variability, particularly with HistGB dropping to 0.93 accuracy and 0.85 recall, indicating sensitivity to classifier choice. Xception-AdamW is clearly the weakest model, recording the lowest scores overall—test accuracy falls to 0.90 with DT and HistGB, precision dips to 0.83, and recall to 0.80, though specificity remains high at 1.00.

**Table 7:** Test accuracy of Various machine learning classifiers on the extracted deep features. (Values in %).

**Model**	**Metrics**	**RF**	**DT**	**LR**	**HistGB**	**XGB**
DenseNet201-AdamW	Test accuracy	0.97	0.97	0.97	0.97	0.97
	Precision	0.95	0.95	0.95	0.95	0.95
	Recall	0.95	0.95	0.95	0.95	0.95
	Specificity	1.00	1.00	1.00	1.00	1.00
InceptionResNetV2-AdamW	Test accuracy	0.97	0.97	0.95	0.95	0.95
	Precision	0.95	0.95	0.91	0.91	0.91
	Recall	0.95	0.95	0.90	0.90	0.90
	Specificity	1.00	1.00	1.00	1.00	1.00
InceptionV3-AdamW	Test accuracy	0.95	0.97	0.95	0.93	0.95
	Precision	0.91	0.95	0.91	0.87	0.91
	Recall	0.90	0.95	0.90	0.85	0.90
	Specificity	1.00	1.00	1.00	1.00	1.00
ResNet101V2-RMSprop	Test accuracy	0.97	0.97	0.97	0.97	0.97
	Precision	0.95	0.95	0.95	0.95	0.95
	Recall	0.95	0.95	0.95	0.95	0.95
	Specificity	1.00	1.00	1.00	1.00	1.00
Xception-AdamW	Test accuracy	0.93	0.90	0.93	0.90	0.90
	Precision	0.87	0.86	0.87	0.83	0.83
	Recall	0.85	0.85	0.85	0.80	0.80
	Specificity	1.00	0.95	1.00	1.00	1.00
DenseNet169-AdamW	Test accuracy	0.97	0.95	0.97	0.95	0.97
	Precision	0.95	0.91	0.95	0.91	0.95
	Recall	0.95	0.90	0.95	0.90	0.95
	Specificity	1.00	1.00	1.00	1.00	1.00

**Figure 12:**
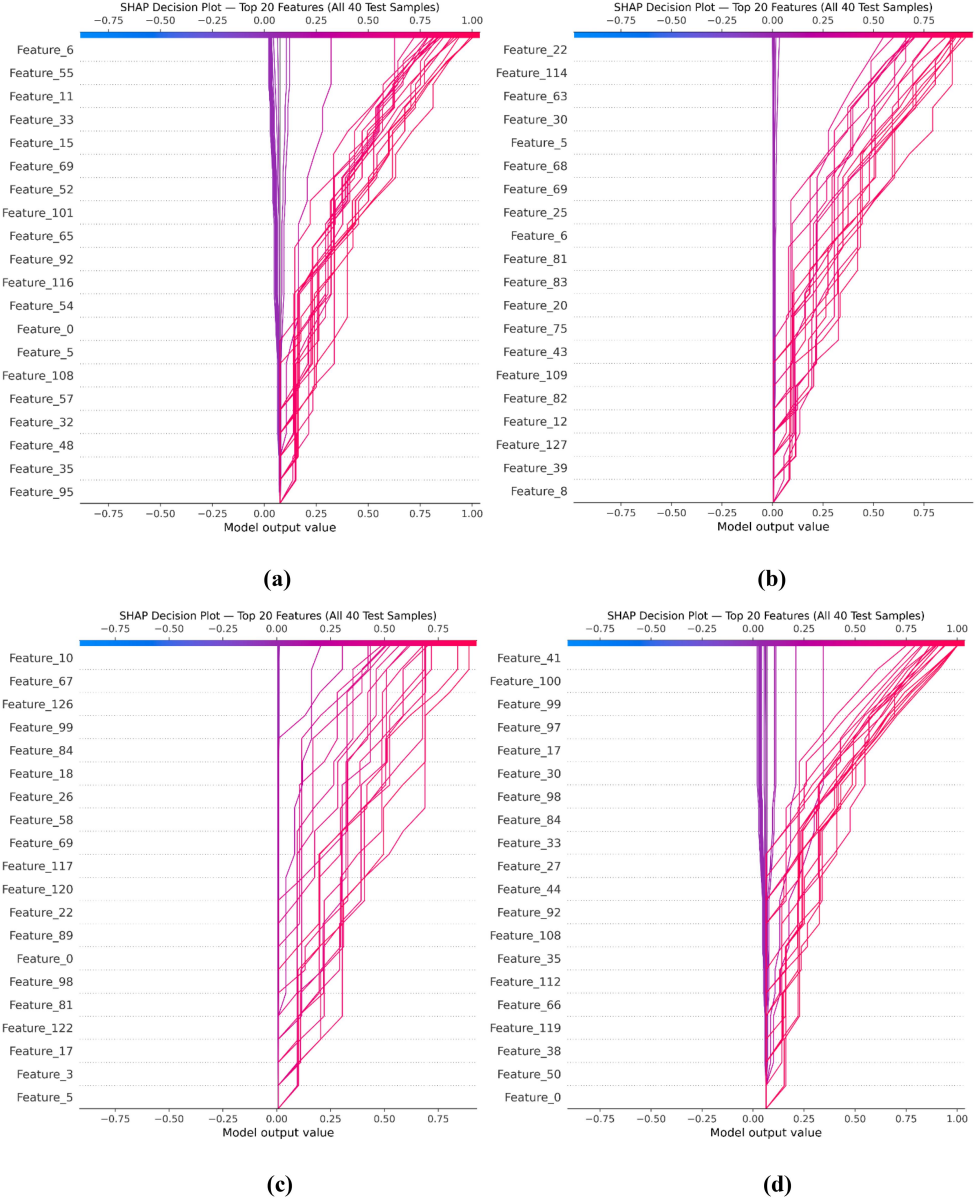
SHAP decision plots of: **(a)** InceptionV3-adamW; **(b)** DenseNet201-RMSprop; **(c)** DenseNet169-adamW; **(d)** ResNet101V2-RMSprop.

Figure 12 shows SHAP decision plots for four model-optimizer pairs. InceptionV3-AdamW (a) exhibits stable positive shifts toward pes planus. DenseNet201-RMSprop (b) shows sharper decision paths, indicating dominance by a few features. DenseNet169-AdamW (c) demonstrates gradual accumulation of multiple features. ResNet101V2-RMSprop (d) achieves the clearest separation with strong positive contributions.

**Figure 13:**
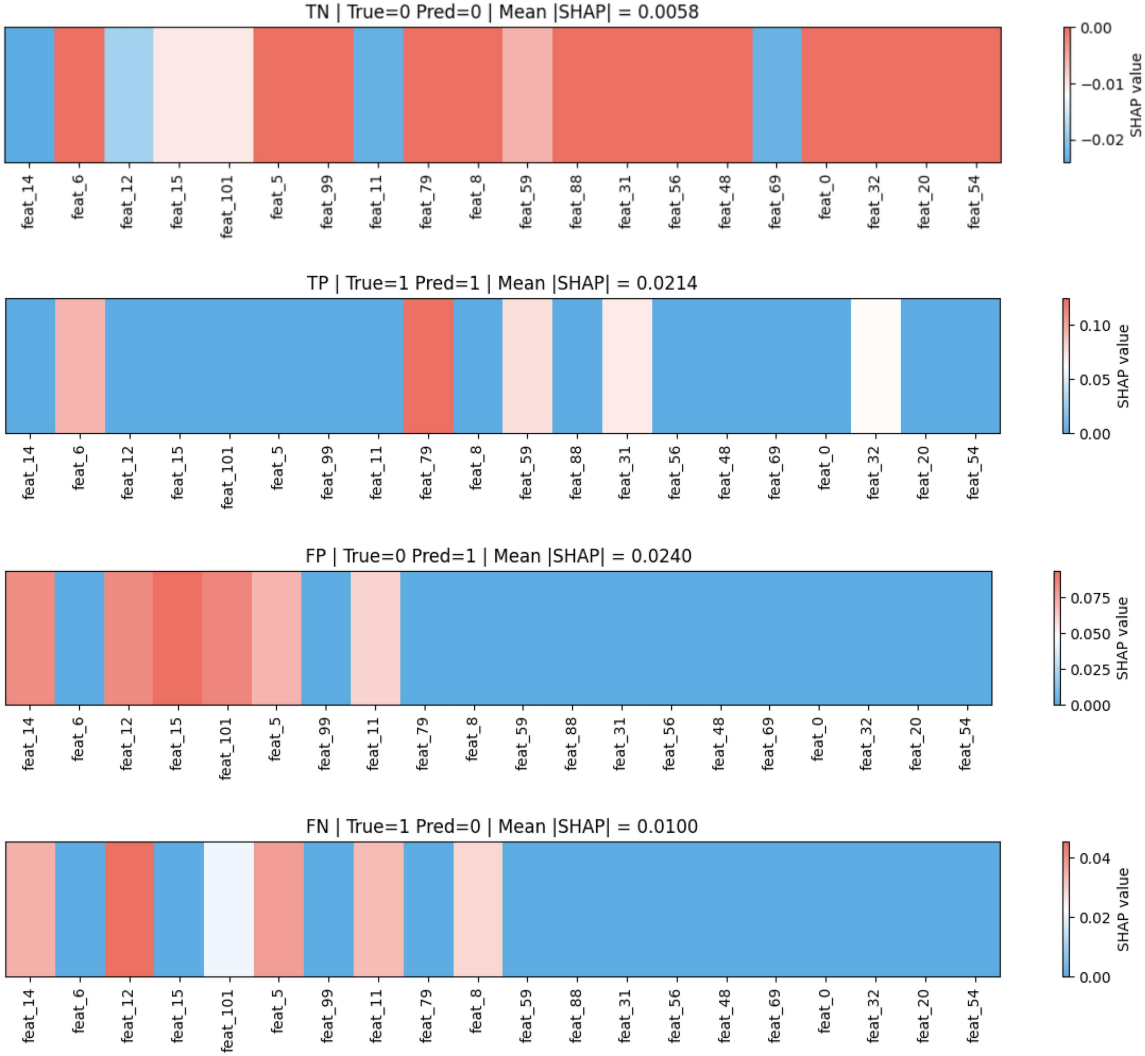
SHAP-Based explainability analysis for four outcome categories.

The SHAP heatmaps as in Figure 13 provide prediction-level interpretability across four outcome categories. For True Positives (correctly predicted Pes Planus), feat_79 and feat_14 show the strongest positive SHAP contributions, meaning they reliably push the model toward the correct positive prediction. In False Positives (healthy cases misclassified as Pes Planus), feat_14, feat_6, feat_12, and feat_15 dominate with high positive SHAP values, indicating these features misled the model. For True Negatives, the SHAP values are predominantly negative (red in the flipped scale), with feat_6, feat_11, feat_99, and feat_0 suppressing the positive prediction correctly. In False Negatives, mixed signals from feat_6 and feat_12 suggest conflicting feature contributions caused the model to miss actual Pes Planus cases.

## 4 Discussion

This study compared three approaches for pes planus classification using x-ray images: handcrafted LBP features with ML classifiers (best: Random Forest, 72% accuracy), end-to-end deep learning (best: ResNet101V2-RMSprop, 92.86% ± 2.82% mean accuracy, 98.21% best single-run accuracy), and deep feature extraction followed by ML classification (best: ResNet101V2-RMSprop features + Decision Tree, 97% accuracy, 95% recall for pes planus, and 100% specificity). SHAP-based explainability revealed consistent feature importance patterns, though latent features remain to be mapped to anatomical landmarks.

### 4.1 Comparison with previous x-Ray-based studies

When compared with x-ray-based deep learning studies for pes planus, our results are competitive. Danaci et al. (6) reported 97% accuracy using feature selection with XGBoost on x-ray images. Kokulu et al. (7) achieved 98.02% accuracy using DenseNet-201 with transfer learning. Dogan et al. (3) reported 96.8% F1-score using a CNN-ViT hybrid. Koo et al. (9) developed a deep learning tool to improve reproducibility of radiographic angle measurement. Our best end-to-end model (ResNet101V2-RMSprop) achieved 92.86% mean accuracy and 98.21% best-run accuracy, while our hybrid approach (deep features + Decision Tree) achieved 95% recall for pes planus. Direct performance comparisons across studies are limited by different datasets, class distributions, and validation strategies.

### 4.2 Clinical utility and proposed role

The proposed system is intended as a clinical decision support system (CDSS) for radiologists and orthopedic specialists, not as a replacement for human diagnosis. In practice, the model could: (a) flag suspicious x-ray images for priority review, (b) provide SHAP-based visual explanations to justify its classification, (c) reduce inter-observer variability in arch angle interpretation, and (d) serve as a training tool for residents. Even with standard weight-bearing x-rays, manual angular measurement has moderate inter-rater reliability; an automated, explainable model can offer consistent second-opinion support.

### 4.3 Stability and generalization

The better stability seen with trained models matches earlier results that show how weighted decay-based optimization and root mean square based optimization improves feature consistency and reduces gradient noise. The model demonstrates strong generalization across the validation split held back for internal use and the held-out separate test set. However, we still need to validate the model across different populations and imaging setups.

### 4.4 Limitations

Despite strong performance, this study has several limitations. First, the dataset is single-center and moderately sized, raising potential overfitting concerns despite Monte Carlo validation. While Monte Carlo cross-validation reduces split-dependent bias, the relatively small test set (56 images) means that reported accuracy may still be optimistic. Future work should validate these results on a larger, independent multi-center dataset. Second, external validation on an independent multi-center dataset was not performed because no such publicly available pes planus x-ray dataset currently exists. Generalization to other populations, x-ray systems, or imaging protocols therefore remains to be established as future work when such data become available. Third, the dataset includes only single static lateral x-ray images and does not incorporate multi-view or dynamic weight-bearing information, which may capture additional arch characteristics. Fourth, while focal loss and augmentation partially addressed class imbalance, residual bias cannot be ruled out. Fifth, the study lacks multiple angles or complementary imaging methods, which may limit the model's ability to capture the full three-dimensional details of arch collapse. Larger, multi-center datasets are needed to confirm the generalizability and strength of the proposed fusion framework.

### 4.5 Clinical interpretation of SHAP-identified features

While the SHAP analysis in this study identifies latent feature indices (e.g., feat_100, 99, 41, 84) rather than directly mappable anatomical landmarks, Grad-CAM provides spatial localization to validate these features.

**Figure 14:**
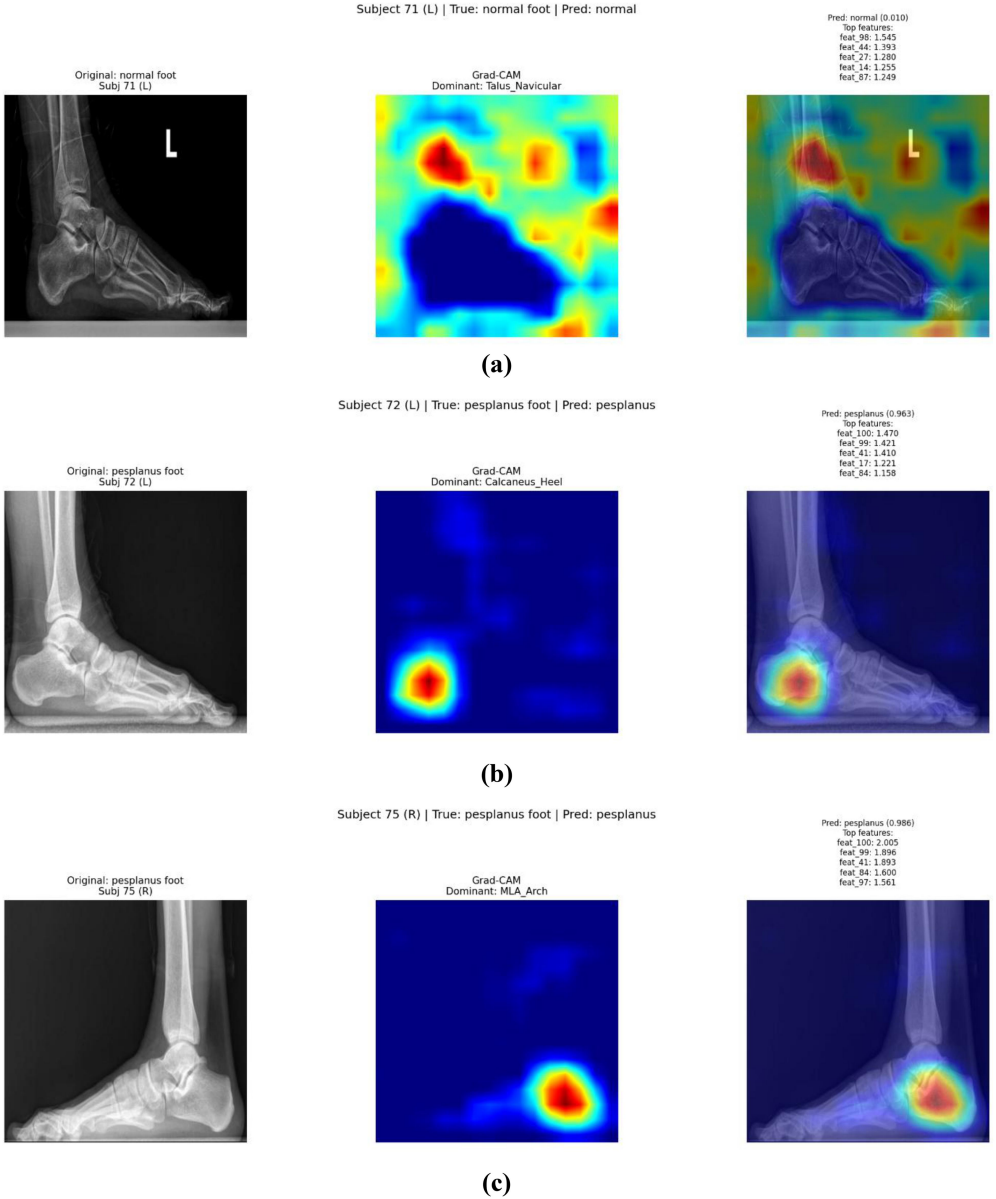
Grad-CAM visualizations for pes planus and normal feet using the ResNet101V2-RMSprop model. **(a)** Normal foot (Subject 71L) showing attention on the talus-navicular region with top features feat_98, 44, 27, 14, 87. The model consistently focuses on clinically relevant anatomical structures: MLA and calcaneus for pes planus, and talus-navicular for normal feet. **(b)** Pes planus foot (Subject 72L) showing attention on the calcaneal region with top features feat_100, 99, 41, 17, 84. **(c)** Pes planus foot (Subject 75R) showing dominant attention on the medial longitudinal arch (MLA) with top features feat_100, 99, 41, 84.

The Grad-CAM visualizations in Figure 14 provide spatial validation of the SHAP feature importance analysis. For pes planus feet (Figures 14b,c), the model's attention is concentrated on two clinically critical regions: the medial longitudinal arch (MLA) and the calcaneus (heel). The MLA is the primary anatomical structure affected in flatfoot deformity, where arch collapse leads to the characteristic flattening. The calcaneus is relevant due to its role in hindfoot alignment and the measurement of calcaneal inclination angle (<18° defines pes planus). The top contributing features for these pes planus images (feat_100, 99, 41, 84) align perfectly with the SHAP-identified discriminative features.

For normal feet (Figure 14a), the model's attention shifts to the talus-navicular region, which is clinically appropriate as normal foot biomechanics involve proper talar alignment and navicular position. The top features for normal classification (feat_98, 44, 27, 14, 87) differ from those dominating pes planus classification, confirming that the model has learned class-specific feature representations.

These findings directly address the clinical interpretation of SHAP-identified features, demonstrating that the most influential latent features correspond to anatomically meaningful regions: the MLA, calcaneus, and talus-navicular complex. This bridges the gap between latent feature importance and clinically interpretable anatomy, enhancing the clinical utility of the proposed system.

The features highlighted in Figure 13 represent global SHAP importance across all test samples, while those discussed here are specific to individual Grad-CAM visualizations. While Grad-CAM provides spatial localization linking latent features to anatomical regions, the features identified in SHAP decision plots (Figure 13) and those highlighted in Grad-CAM visualizations represent complementary perspectives from different analysis modalities. Establishing a direct one-to-one mapping between specific feature indices and precise anatomical landmarks remains an ongoing challenge and is a direction for future investigation.

## 5 Conclusion

This study compared three classification strategies for pes planus: handcrafted LBP features (best: Random Forest, 72% accuracy), end-to-end deep learning (best: ResNet101V2-RMSprop, 92.86% ± 2.82% accuracy), and deep feature extraction followed by ML classification (best: ResNet101V2-RMSprop features + Decision Tree, 97% accuracy, 95% recall, 100% specificity). SHAP and Grad-CAM confirmed that the model focuses on clinically relevant regions: the medial longitudinal arch and calcaneus for pes planus, and the talus-navicular region for normal feet. The hybrid approach outperformed both pure handcrafted features and some end-to-end models in recall and specificity, making it suitable for clinical screening. Future work will focus on external validation and multi-view imaging.

## Data Availability

The original contributions presented in the study are included in the article/Supplementary Material, further inquiries can be directed to the corresponding author.
